# Genome-Wide Analysis of NAC Transcription Factor Gene Family in *Morus atropurpurea*

**DOI:** 10.3390/plants14081179

**Published:** 2025-04-10

**Authors:** Yujie Yang, Meiyu He, Kaixin Zhang, Zeyang Zhai, Jialing Cheng, Yue Tian, Xu Cao, Li Liu

**Affiliations:** 1 Jiangsu Key Laboratory of Sericultural and Animal Biotechnology, School of Biotechnology, Jiangsu University of Science and Technology, Zhenjiang 212100, China; yang1344813698@163.com (Y.Y.); hemy0504@163.com (M.H.); zhangkaixin202202@163.com (K.Z.); zhai98doc@163.com (Z.Z.); chengjialingl@yeah.net (J.C.); shikougiushan@163.com (Y.T.); 2 Key Laboratory of Silkworm and Mulberry Genetic Improvement, Ministry of Agriculture and Rural Affairs, Sericultural Scientific Research Center, Chinese Academy of Agricultural Sciences, Zhenjiang 212100, China

**Keywords:** mulberry, NAC gene family, expression pattern, drought stress, fungal disease

## Abstract

The NAC (NAM/ATAF1/2/CUC2) transcription factors are pivotal regulators in plant development and stress responses. Despite the extensive studies on the NAC gene family across various plant species, the characterization of this gene family in mulberry (*Morus atropurpurea*) remains unexplored. Here, we conducted a genome-wide identification and characterization of the NAC gene family in *M. atropurpurea*. A total of 79 *MaNAC* genes were identified and classified into 20 subgroups, displaying an uneven distribution across the 14 chromosomes. The structural analysis found that most *MaNAC* genes possess at least three exons and contain the conserved NAC domain and characteristic motifs at the N-terminus. Eleven collinear gene pairs were identified in *M. atropurpurea* genome. Interspecies collinearity analysis demonstrated a closer evolutionary relationship between *M. atropurpurea* and *Populus trichocarpa*, supported by the identification of 116 collinear gene pairs. Expression profiling revealed dynamic changes in the transcript levels of most *MaNAC* genes during mulberry fruit maturation. Notably, the eight MaNAC members from the OsNAC7 subfamily exhibited tissue-specific expression patterns. A significant proportion of *MaNAC* genes displayed varying degrees of responsiveness to drought stress and sclerotium disease. *MaNAC12*, *MaNAC32*, *MaNAC44* and *MaNAC67* emerged as the most highly responsive candidates. Overexpression of *MaNAC69* enhanced drought tolerance in *Arabidopsis*. These findings provide a robust foundation for future functional studies and mechanistic investigations into the roles of the NAC gene family in *M. atropurpurea*, offering insights into their contributions to development and stress adaptation.

## 1. Introduction

Mulberry (*Morus* spp.) is a fundamental tree species of the global sericulture industry, with its leaves serving as the primary food source for silkworms (*Bombyx mori*). Mulberry fruits are rich in vitamins, minerals and bioactive compounds such as anthocyanins, flavonoids and DNJ (1-deoxynojirimycin), offering a wide range of health benefits [1]. Beyond its economic importance, mulberry also holds significant ecological values in afforestation of degraded lands, phytoremediation of polluted soils, conservation of soil and water and carbon sequestering, making it an ideal plant for sustainable development [2]. However, the increasing occurrences of extreme climate such as drought, heat and cold have led to substantial economic loss in mulberry plantations. Additionally, fruit sclerotiniose, a prevalent fungal disease in fruit-bearing mulberry varieties caused by *Ciboria shiraiana*, severely affects fruit quality and downstream utilization [3]. Therefore, functional studies on genes associated with mulberry stress responses are of great significance for the sustainable development of the sericulture industry.

Plant transcription factors (TFs) are a class of essential proteins that regulate various biological processes. The NAC proteins constitute one of the largest families of plant-specific TFs, playing critical roles in plant growth, development and stress responses. The family name “NAC” is derived from three transcription factors, namely NAM (*No Apical Meristem* from *Petunia hybrida*), ATAF1/2 (*Arabidopsis* transcription activation factor) and CUC2 (*cup-shaped cotyledon* from *Arabidopsis thaliana*), all of which share a similar DNA-binding domain [4]. Structurally, NAC proteins are characterized by a highly conserved N-terminal NAC domain responsible for DNA binding and dimerization, and a variable C-terminal transcriptional regulatory region that often confers functional specificity [5]. The NAC domain, typically comprising approximately 150 amino acids, is divided into five subdomains (A–E). Subdomains A and B are primarily involved in DNA binding and dimerization, which are essential for transcriptional activity [6]. Subdomains C, D and E contribute to protein–protein interactions, nuclear localization and functional specificity, respectively [7,8].

Over the past decade, the NAC gene family has been extensively characterized in various plant species, such as *A. thaliana* and *Oryza sativa* [5,9], *Mattis domestica* [10], *Broussonetia papyrifera* [11], *Ginkgo biloba* [12], *Populus tomentosa* [13] and *Eucommia ulmoides* [14]. Increasing research demonstrates the pivotal roles of NAC TFs in plant growth, development and responses to biotic and abiotic stresses. In *A. thaliana*, *CUC1* and *CUC2* are essential for the formation of boundary regions in the shoot apical meristem [4]. Moreover, NAC TFs have been shown to activate ripening-related alcohol acyltransferase, which catalyzes the formation of volatile ester in multiple fruit species [15]. In poplar, wood-associated NAC TFs (PtrW*NDs*) act as master regulators of secondary wall biosynthesis during wood formation [16]. In *Arabidopsis*, the overexpression of *ANAC019*, *ANAC055* and *ANAC072* enhanced drought tolerance by activating the stress-responsive gene *ERD1* [17]. Similarly, the rice *ONAC022* gene conferred increased salt and drought tolerance through an ABA-mediated pathway [18]. In apple, *MdNAC104*-overexpressing transgenic apple plants exhibited higher cold tolerance via C-repeat binding factor (CBF)-dependent and independent pathways [19]. Furthermore, NAC TFs are central components of many aspects of a plant’s innate immune system [20]. For instance, *Botrytis cinerea* susceptibility gene *WRKY52* in grapevine was directly regulated by NAC61, which primarily plays a key role in berry ripening progression [21]. Up to now, the characterization of the *NAC* gene family in mulberry was only reported in the wild germplasm *M. notabilis* [22]. Furthermore, stress responses of the NAC gene family in response to biotic or abiotic stresses and their functional validation were not documented in this publication. 

*Morus atropurpurea* cv. Zhongshen1 is an elite cultivar distinguished for its dual-purpose utilizations in both fruit and foliage production. However, it is relatively drought-sensitive [23] and susceptible to fruit sclerotiniose [24]. Recently, the haplotype-resolved chromosomal-level genome assembly of this cultivar has been published [25], providing a valuable resource for in-depth exploration of the NAC gene family. In this work, we aimed to perform a comprehensive genome-wide characterization of *NAC* genes in *M. atropurpurea* and identify candidate *MaNACs* involved in drought and sclerotiniose responses. Specifically, phylogenetic relationships, inter-species collinearity, conserved domain and motifs, gene structure, promotor cis-element and stress profiling were analyzed. Noteworthily, we focused on the members of the OsNAC7 subfamily for their important roles in xylem formation, which further affects water transport efficiency in woody trees [16]. Due to this, the tissue expression profiling, subcellular localization and functional validation under drought stress of two representative members from this subfamily were performed. These results will provide an important basis for elucidating the molecular mechanisms of NAC TFs in regulating mulberry development and stress tolerance, thus supporting future molecular-assisted breeding in mulberry.

## 2. Results

### 2.1. Identification and Phylogenetic Analysis of NAC Gene Family

**Figure 1:**
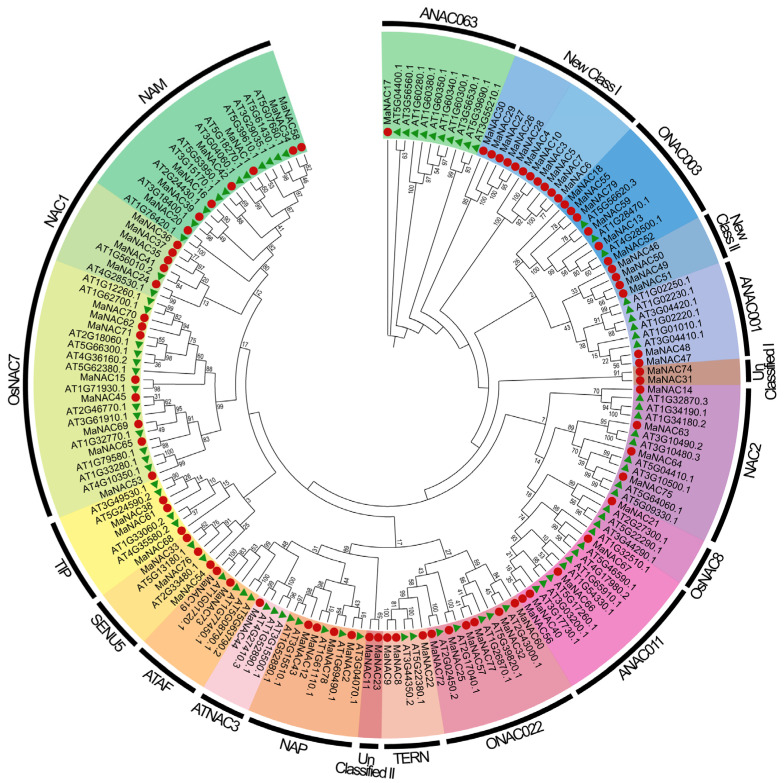
Phylogenetic tree analysis of the *NAC* gene family of *M. atropurpurea* (red solid circle) and *A. thaliana* (green solid triangle). The values on the branches represent bootstrap values ranging from 0 to 100. Different subfamilies are indicated with different highlighted colors.

The NAC domain was utilized as a query to search for MaNAC protein sequences using HMMER software (version 3.3.2), resulting in the identification of 79 non-redundant putative MaNACs, less than the number found in the *A. thaliana* genome. To investigate the evolutionary relationships of the MaNAC proteins, we constructed a phylogenetic tree using NAC protein sequences from *A. thaliana* and *M. atropurpurea*. The AtNACs were classified into 16 subgroups, whereas the MaNAC genes were divided into 20 subgroups including two novel classes and two unclassified subfamilies (Figure 1). The distribution of MaNAC members across subfamilies varied significantly. The largest subfamily, New Class I, comprised 11 MaNAC genes with no homologs in *A. thaliana*. OsNAC7 and NAM represented the second- and third-largest subgroups, with eight and seven MaNAC members. ATNAC3 and OsNAC8 were the smallest subgroups, each containing only one MaNAC member.

**Figure 2:**
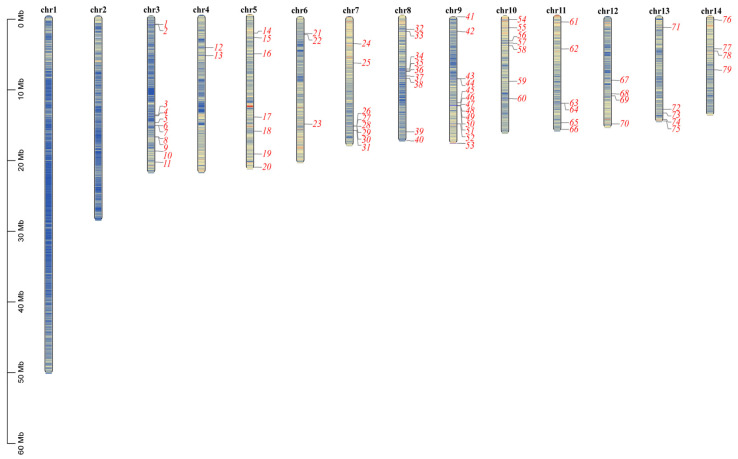
Schematic presentations for the distribution of *MaNAC* genes in *M. atropurpurea* chromosomes. The numbers along each chromosome are short for the names of *MaNAC* genes. The lines inside the chromosomes represent gene density. The scale is provided in megabase (Mb).

The *MaNAC* genes are unevenly distributed across the 14 *M. atropurpurea* chromosomes. No *NAC* genes were localized in chromosomes 1 and 2, and the highest proportion of NAC genes were localized on chromosomes 3 and 9, accounting for 30% (Figure 2, Table S1). Each mulberry gene was named *MaNAC01* to *MaNAC79* according to its chromosomal position. All MaNAC proteins contain the NAM domain (PF02365). We examined the physicochemical properties of the 79 MaNAC genes, including amino acid number, molecular weight, isoelectric point (pI) and predicted subcellular localization (Table S1). The amino acid length ranged from 128 (MaNAC30) to 772 (MaNAC68), and the relative molecular weight varied from 14.9 kDa (MaNAC30) to 86.0 kDa (MaNAC68), showing a positive correlation with the amino acid length. The pI values spanned from 4.56 (MaNAC64) to 10.13 (MaNAC35), with 28 genes encoding basic proteins (pI > 7) and the rest encoding acidic proteins (pI < 7). All MaNAC proteins were predicted to localize to the nucleus.

### 2.2. Conserved Protein Motif and Gene Structure Analysis of MaNACs

**Figure 3:**
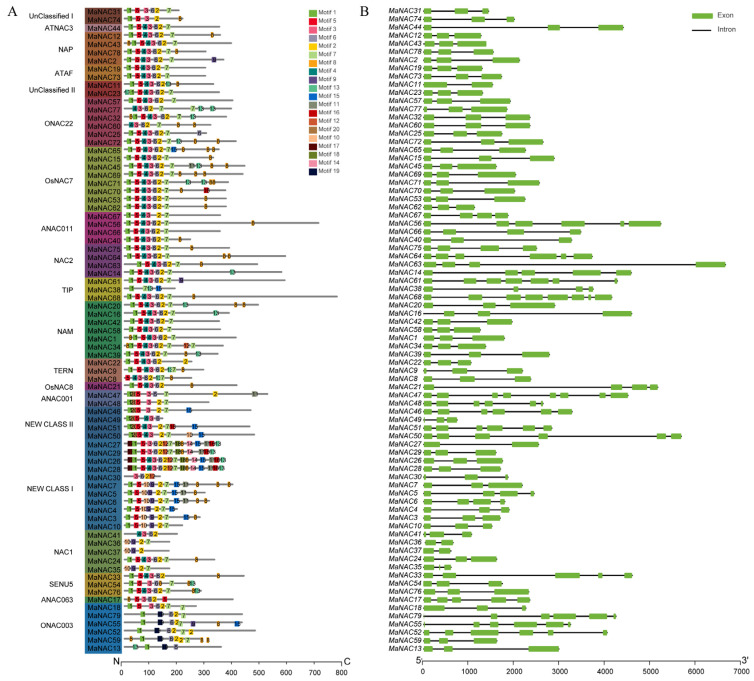
Conserved motifs (**A**) and exon-intron structures (**B**) of *MaNAC* genes. The motifs, numbered from 1 to 20, are displayed in different colored boxes.

The NAC domain responsible for DNA binding plays a crucial role in the biological functions of NAC proteins. The multi-sequence alignment showed that the NAC domain can be divided into five subdomains A–E. Most MaNAC proteins contain all the subdomains within their amino terminus (Figure S1). However, subdomains A, B, C or E were entirely absent in *MaNAC35*, *36*, *37*, *38*, *30* and *49*, while *MaNAC8*, *41* and *77* exhibited incomplete subdomains (Figure S1). A total of 20 conserved motifs were identified in MaNAC proteins (Figure 3A), with lengths ranging from 28 to 50 amino acids (Figure S2). Motifs 1–6 were found to approximately corresponded to subdomains A–E. Notably, Motif 19 is exclusively present in the ONAC003 subfamily, suggesting its potential role in functional diversification within this subgroup. 

To gain further insights into the structural organization of *MaNAC* genes, we examined their intron/exon compositions (Figure 3B). The analysis showed that the number of introns varied from one to seven and the number of exons ranged from two to eight. MaNAC47 and MANAC68 exhibited the most complex structures, containing the largest number of introns (7) and exons (8). The majority of MaNAC genes were found to possess three exons. Notably, genes within the same subfamilies displayed similar exon/intron patterns. For instance, the NAC2 subfamily typically contained 4~6 exons, whereas both NAM and TERN subfamilies consistently showed 3 exons (Table S2).

**Figure 4:**
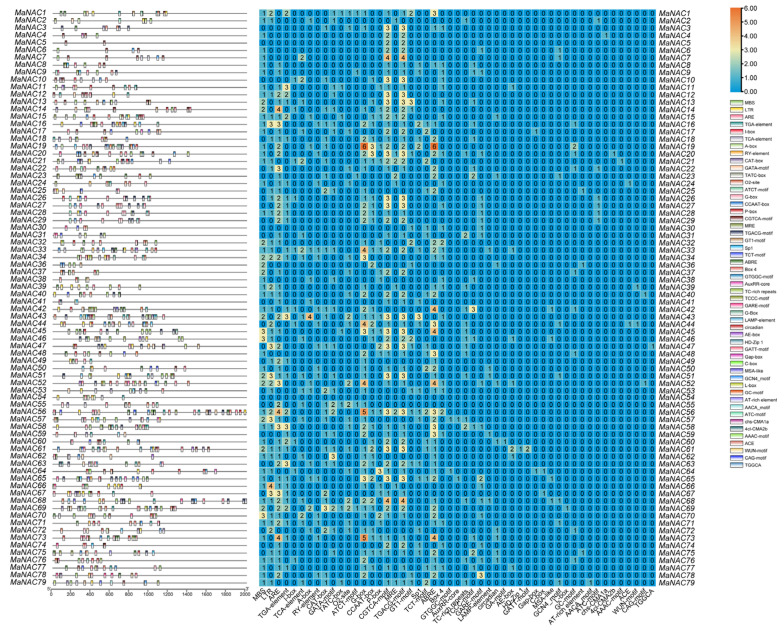
Cis-acting elements within the promoter region (2000 bp upstream of the CDS) of *MaNAC* genes. The squares with different colors on the left represent different types of cis-acting elements and their positions in the promoter region. The number of various cis-acting elements in the promoter region of each *MaNAC* gene is presented on the right.

To investigate the potential regulatory mechanisms, we analyzed the 2000 bp upstream of the *MaNAC* genes’ transcription start site using the PlantCARE database (Figure 4, Tables S3 and S4). The analysis revealed a significant prevalence of cis-elements associated with light responses, methyl jasmonate signaling and anaerobic stress responses. Specifically, we identified 87, 53 and 48 cis-elements related to abscisic acid, gibberellin and auxin responses, respectively. Furthermore, 61 elements were associated with low temperature response, while 72 elements were linked to drought stress response. 

### 2.3. Gene Duplication and Collinearity Analysis of MaNACs

**Figure 5:**
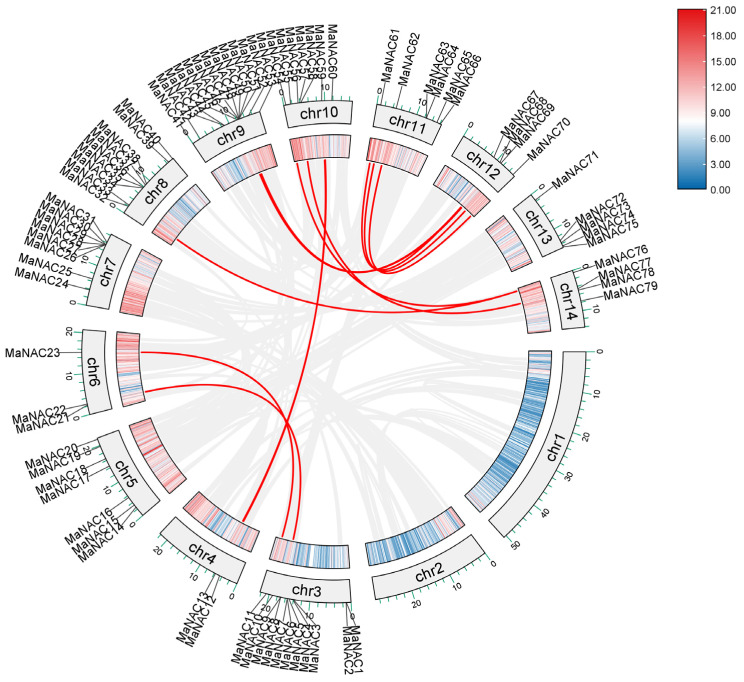
Intraspecies collinearity of the *MaNAC* gene family. The red lines represent duplication events of *MaNAC* genes. The color code from blue to red indicates gene density from 0 to 21.

**Figure 6:**
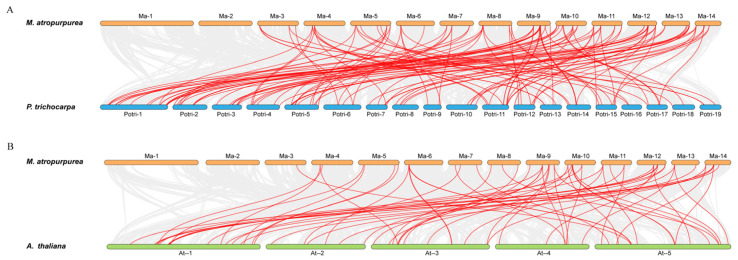
Synteny analysis of *NAC* genes in *M. atropurpurea* with *P. trichocarpa* (**A**) and *A. thaliana* (**B**). The red lines highlight the collinear *NAC* gene pairs.

To investigate the role of gene duplication in the expansion of the MaNAC family expansion, we annotated and analyzed the intraspecific collinearity of *MaNAC* genes. The analysis revealed 11 collinearity pairs within the MaNAC gene family (Figure 5). Chromosomes 11 and 12 harbored the highest number of duplicated genes, with three pairs each. The remaining three paralogous gene pairs were located on chromosomes 3 and 6, chromosomes 9 and 12, and chromosomes 10 and 14. Additionally, a single segmental duplication was identified on chromosomes 8 and 14. To further explore the evolutionary interrelationships of the *NAC* genes across different species, we performed interspecies collinearity analysis between *M. atropurpurea* and *A. thaliana* or *P. trichocarpa* (Figure 6). The results revealed a closer evolutionary relationship between *M. atropurpurea* and *P. trichocarpa*, with a total of 116 collinear pairs identified for NAC family members. In contrast, 63 collinear gene pairs were found between *M. atropurpurea* and *A. thaliana*. 

### 2.4. Temporal Expression and Response Profiling of MaNACs

**Figure 7:**
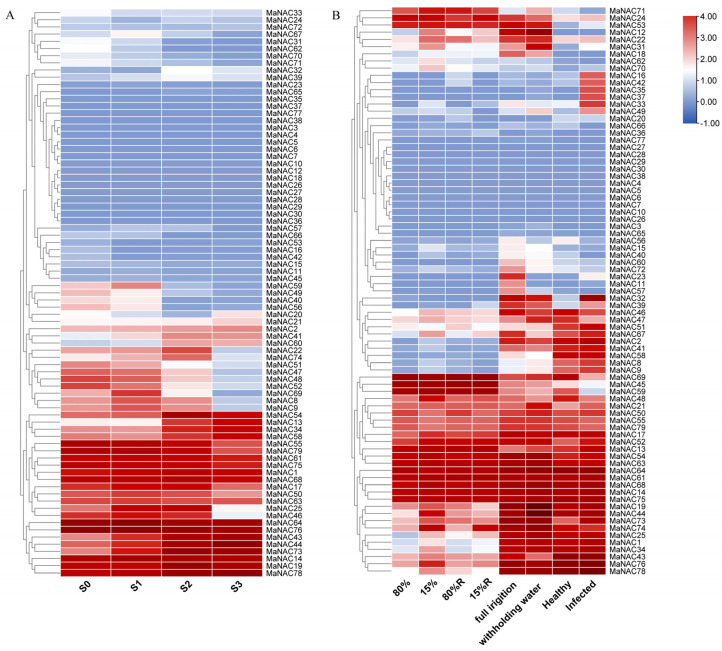
The temporal expression profile of *MaNAC* gene family in different stages during mulberry fruit maturation (**A**); the response profiles of *MaNAC* gene family to drought, rewatering or sclerotinose infection in cambium, roots and fruits using transcriptome (**B**). The values of log_2_(FPKM + 1) are presented in the heatmap. S0: inflorescence; S1: green fruits; S2: reddish fruits; S3: purple fruits; 80%: 80% of the SSWC (soil saturated water content); 15%: 15% of the SSWC; 80%R and 15%R: rewatering to 80% of the SSWC.

During mulberry fruit maturation, 41 *MaNAC* genes demonstrated relatively higher expression levels, 18 were expressed at low levels and the remaining ones showed no detectable expression (Figure 7A). The expression levels of several genes, including *MaNAC22*, *MaNAC74*, *MaNAC51*, *MaNAC47*, *MaNAC48*, *MaNAC52*, *MaNAC69*, *MaNAC8* and *MaNAC9*, decreased sharply upon reaching the purple-fruit stage (S3). Conversely, other genes, including *MaNAC54*, *MaNAC13*, *MaNAC34* and *MaNAC58*, showed a dramatic increase in transcript levels as the fruit transitioned from the green (S1) to the red stage (S2).

Transcriptomic data were analyzed under different types of drought stress (prolonged drought, sustained drought and rewatering) and *C. shiraiana* infection (Figure 7B). Most *MaNAC* genes were expressed in mulberry stem cambium, among which 13 *MaNAC* genes, namely *MaNAC12*, *MaNAC25*, *MaNAC33*, *MaNAC34*, *MaNAC44*, *MaNAC48*, *MaNAC52*, *MaNAC67*, *MaNAC68*, *MaNAC70*, *MaNAC74*, *MaNAC76* and *MaNAC78*, were drought responsive, showing upregulation in response to 15% of soil water content. The expression of these genes returned to levels comparable to the control after rewatering, except for *MaNAC74*, which maintained significantly higher expression. Overall, *MaNAC* genes in roots exhibited higher transcripts compared to those in the stem cambium. A distinct set of drought-responsive *MaNAC* genes was identified in roots under water deficit conditions. Specifically, eight genes, namely *MaNAC12*, *MaNAC19*, *MaNAC21*, *MaNAC31*, *MaNAC44*, *MaNAC47*, *MaNAC49* and *MaNAC71*, were significantly upregulated, whereas seven genes, *MaNAC11*, *MaNAC23*, *MaNAC43*, *MaNAC56*, *MaNAC57*, *MaNAC67* and *MaNAC72*, were significantly downregulated. The overlapping genes *MaNAC12*, *MaNAC44* and *MaNAC67* between cambium and roots may indicate their role in functional diversity. In response to fruit sclerotinose, a larger number of *MaNAC* genes were responsive in mulberry fruits, with 15 upregulated and 5 downregulated. Notably, *MaNAC32* exhibited an approximately 200-fold change, suggesting that it could play an important role in the response to *C. shiraiana* infection. Additionally, the expressions of 14 *MaNAC* genes, namely *MaNAC3–7*, *MaNAC10*, *MaNAC26–30*, *MANAC38*, *MaNAC65* and *MaNAC77*, were not detected in the transcriptome data across any tissues or stress conditions.

### 2.5. Tissue-Specific Expression Profiling of OsNAC7 Subfamily

**Figure 8:**
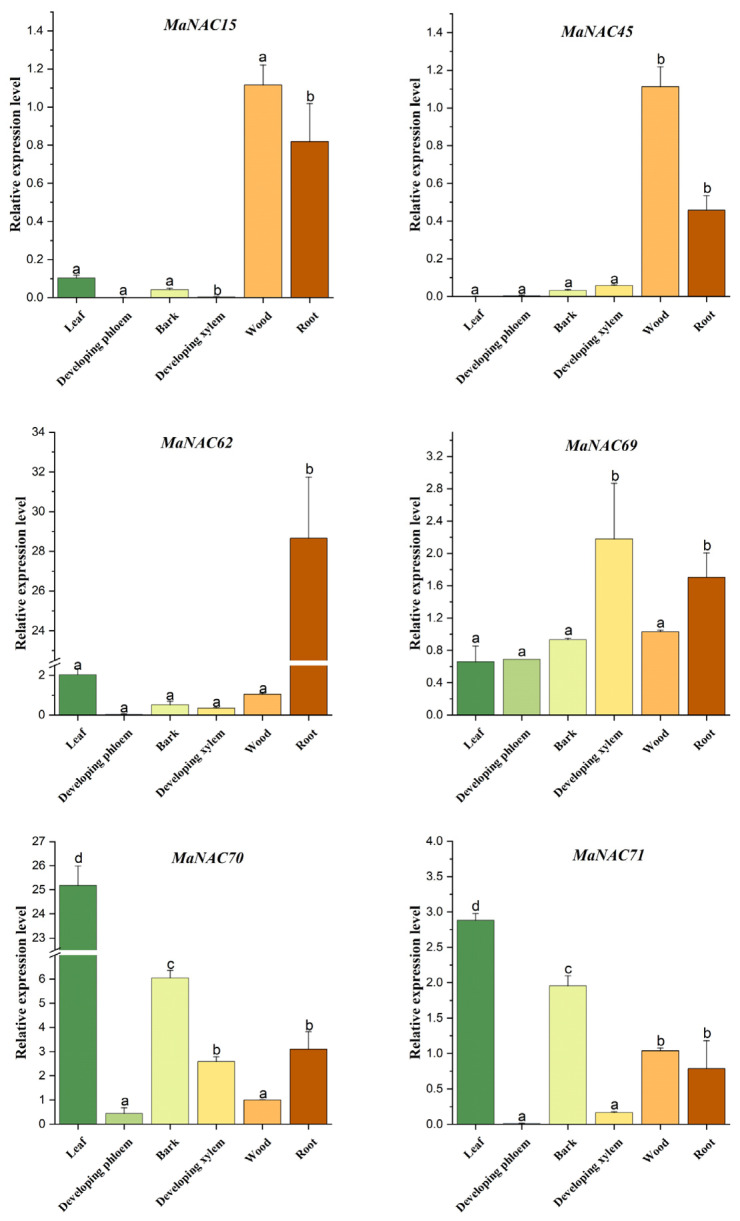
Tissue-specific expression patterns of the six members from OsNAC7 based on qRT-PCR with the transcript levels in wood defined as 1. The bar indicates mean ± SE (n = 6). Different letters on the bars indicate significant differences at *p* < 0.05.

We analyzed the relative transcriptional expression levels of the OsNAC7 subfamily across six distinct tissues: leaf, developing phloem, bark, developing xylem, wood and root (Figure 8). *MaNAC15* and *MaNAC45* were predominantly expressed in wood and root. *MaNAC62* displayed significantly higher transcriptional levels in roots, while *MaNAC69* was expressed in all examined tissues with notably higher transcripts in the developing xylem and roots. In contrast, *MaNAC70* and *MaNAC71* exhibited the highest transcriptional levels in non-woody tissues, such as leaf and bark, and lower levels in root, wood and developing phloem/xylem. Additionally, *MaNAC53* and *MaNAC65* were not detectable in any of these tissues.

### 2.6. Subcellular Localization of MaNAC15 and MaNAC69

**Figure 9:**
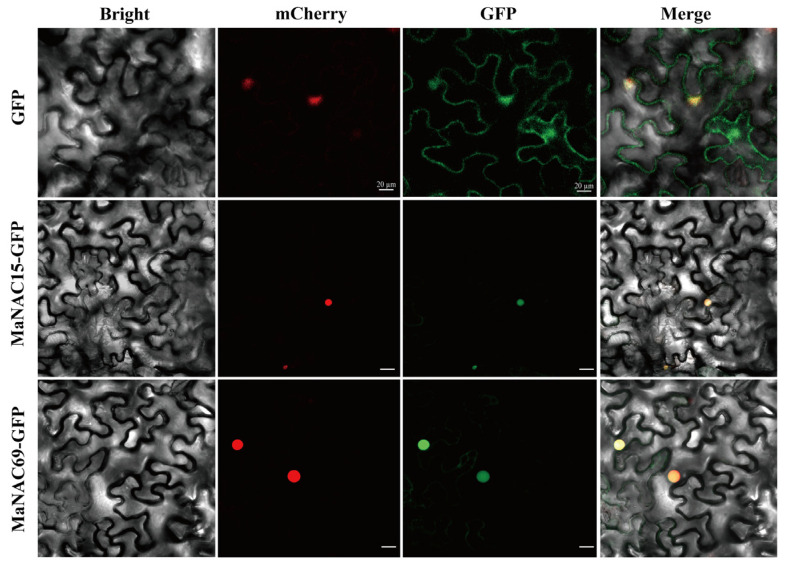
Subcellular location of MaNAC15 and 69 in *M. atropurpurea*. GFP indicates green fluorescence photography; mCherry indicates red fluorescence photography. The scale bar is 20 μm.

To determine the subcellular localization of MaNAC proteins, MaNAC15 and 69 were selected for subcellular localization analysis as they are representative members of the OsNAC7 subfamily. The signal could be detected only in the nucleus when these two genes were co-expressed with GFP (Figure 9), confirming their roles as transcription factors. The nuclear localization of these proteins suggests that they may regulate gene expression by binding to specific DNA sequences in the promoter regions of target genes.

### 2.7. Drought Tolerance of MaNAC69-Overexpressing Arabidopsis 

**Figure 10:**
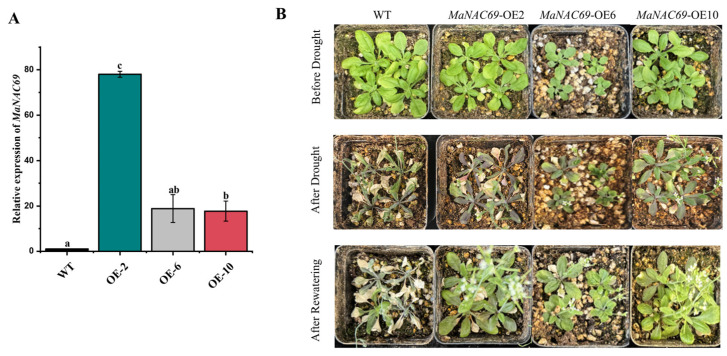
*MaNAC69*-overexpressing *Arabidopsis* in response to drought stress. (**A**) *MaNAC69* expression levels in transgenic *Arabidopsis* lines; (**B**) morphology of transgenic *Arabidopsis* and wild types under drought stress and after rewatering. Wild types were used as CK group. Data are presented as means ± SD of three biological replicates. Different letters on the bars indicate significant differences at *p* < 0.05.

MaNAC69 is homologous to SND1/NST3 in Arabidopsis; therefore, it was selected for functional validation in Arabidopsis. qRT-PCR was conducted to test and validate the overexpression levels of transgenic Arabidopsis plants. The expression levels of the three selected overexpression lines were higher than that of the wild type (Figure 10A). MaNAC69-overexpressing Arabidopsis showed similar growth to that of the wild type (Figure 10B). When exposed to drought stress, MaNAC69-overexpressing Arabidopsis showed enhanced drought stress tolerance after 7 days of drought stress, while the wild type showed significant growth inhibition (Figure 10B). All the transgenic Arabidopsis plants resumed growth after rewatering, but the wild type did not show any recovery after rehydration. 

## 3. Discussion

In this study, 79 *MaNAC* genes were identified in the genome of *M. atropurpurea*. The number of MaNAC genes is less than *A. thaliana* (117) [5] and *P. tomentosa* (270) [13], similar to those of *M. notabolis* [22] and paper mulberry (both 79) [11], also belonging to the Moraceae family. We followed the classification of the NAC family in *Arabidopsis* which divided NAC TFs into 16 subfamilies [5], while two new and two unclassified classes were also identified in the genome of *M. atropurpurea*. This expansion suggests that the NAC gene family in mulberry has undergone significant diversification, potentially driven by species-specific evolutionary pressures. The largest subfamily, New Class I, comprised 11 *MaNAC* genes with no homologs in *A. thaliana*, indicating that these genes may have evolved unique functions in mulberry. Similarly, the presence of two unclassified subfamilies highlights the complexity and divergence of the NAC family in *M. atropurpurea*. Such lineage-specific expansions have been observed in other plant species, such as rice [9] and poplar [26], where NAC genes have expanded to accommodate species-specific adaptations. Corresponding to the increased number of subfamily categories, the members within either subfamily were found to decrease. For instance, the members of OsNAC7 and NAM in mulberry were less than *A. thaliana*, but comparable in *M. atropurpurea* and *M. notabolis*. The NAM subfamily plays an important role in morphogenesis [27], while the OsNAC7 subfamily serves as an essential regulator in xylem development [28]. We identified eight members within this subfamily in mulberry, namely *MaNAC15*, *MaNAC45*, *MaNAC53*, *MaNAC62*, *MaNAC65*, *MaNAC69*, *MaNAC70* and *MaNAC71*. The four MaNAC representatives of VND proteins are GWHPDOOT06918 (*MaNAC71*), GWHPDOOT06676 (*MaNAC70*), GWHPDOOT04184 (*MaNAC62*) and GWHPDOOT14867 (*MaNAC15*), functionally characterized as orthologs of the *Arabidopsis VND1* (At2g18060), *VND4* (At1g12260) or *VND5* (At1g62700) and *VND7* (At1g71930). Similarly, one BRN (bearskin) gene and two NST (NAC secondary wall thickening promoting factor) genes that regulate root cap maturation [29] and secondary wall biosynthesis in fibers of stems [30] in *Arabidopsis* were identified. Such species-specific concerted expansion and contraction of MaNAC TFs should be crucial for the adaptive evolution of mulberry under various environmental conditions [31]. Additionally, the uneven distribution of *MaNAC* genes across the 14 chromosomes corresponded to reports in other plant species such as soybean and tomato [32], where NAC genes are often clustered on specific chromosomes. 

Our analysis revealed that most MaNAC proteins contain the five NAC subdomains (A–E) within their N-terminal regions, which is consistent with previous studies in other plants [7,8]. However, the absence or incompleteness of certain subdomains in some MaNAC proteins, such as MaNAC35, 36, 37, 38, 30 and 49, suggests that these proteins may have evolved distinct functional roles. For instance, MaNAC35–37, which showed a complete absence of subdomains A and B, were not detectable during the process of mulberry fruit maturation. MaNAC30, which had no subdomain A and partial subdomain C, and MaNAC38, missing subdomain C, did not respond to drought or fungal infection in either examined tissue. Such results emphasize that the presence of subdomains A–C may be critical for NAC function by influencing dimerization or DNA binding [33]. The identification of 20 conserved motifs in MaNAC proteins further supports the functional diversification of this gene family. Notably, motif 19 was exclusively present in the ONAC003 subfamily, suggesting its potential role in the functional specialization of this subgroup. Similar findings have been reported in rice, where specific motifs were associated with stress-responsive NAC genes [9]. The conservation of motifs 1–6, which correspond to subdomains A–E, underscores their importance in the structural and functional integrity of NAC proteins.

Gene promoters and their associated cis-acting elements play crucial regulatory roles in gene transcription. Previous studies have documented the importance of cis-acting elements in understanding the regulatory mechanisms underlying the biological functions of genes [20,34]. For instance, AtNAC2, known to positively regulate leaf senescence, also negatively affects primary root development by binding to the *ARF8* and *PIN4* promoters in *Arabidopsis* [35]. Otherwise, two cis-acting elements in the promoter of *TaNAC108-A* in drought-tolerant wheat germplasm can be bound to MYB protein, resulting in enhanced expression of *TaNAC071-A* [36]. In this study, 32 *MaNAC* genes had 41 cis-acting elements associated with MYB proteins, indicating that MYB may be an upstream transcriptional regulator of MaNAC expression. We also found 46 promoters possessing 72 drought response elements and 41 promoters containing 61 low-temperature responsive elements, suggesting that these genes may play a crucial role in drought or cold stress responses, as frequently reported in various plant species [26,37]. Furthermore, we identified 427 cis-acting elements associated with the responsiveness to MeJA, gibberellin, ABA, auxin and salicylic acid in the promoters of 75 *MaNAC* genes. Extensive research has demonstrated that cis-acting elements responsive to phytohormones play a pivotal role in modulating the expression of *NAC* genes. For instance, an ABA-responsive element binding protein 3 (AREB3) involved in the ABA signaling pathway in wheat (*Triticum awstivum* L.) could directly bind to *TaNAC48* promoter and activate the expression of *TaNAC48* and affect drought responses [38]. In rice (*O. sativa*), gibberellin could promote cellulose synthesis by rebuilding the DELLA-NAC signaling cascade [39]. The identification of these cis-elements provides a foundation for future studies aimed at elucidating the regulatory networks controlling *MaNAC* gene expression.

Recent studies have revealed that NAC TFs play important roles during the ripening of fleshy fruits and the development of quality traits [40,41]. The dynamics of various pigment levels such as chlorophyll degradation and anthocyanin accumulation determine specific fruit color at certain developmental stages [42]. In tomato (*Solanum lycopersicum*), SlNAC1 targets the promoter region of phytoene synthase 1 (*SlPSY1*) in the carotenoid biosynthesis pathway and inhibits its expression, thus inhibiting carotenoid accumulation during fruit ripening [43]. The accumulation of anthocyanins in red-fleshed apples during the ripening process was closely associated with the transcriptional upregulation of *MdNAC42*. This NAC transcription factor interacts with MdMYB10, a key positive regulator of anthocyanin biosynthesis, to synergistically enhance anthocyanin production [44]. Additionally, PpNAC1 and PpNAC5 in peach (*Prunus persica*) fruit have pleiotropic effects on fruit taste by activating the transcription of genes related to sugar accumulation and organic acid degradation [45]. In this study, the dramatic increase in transcript levels of *MaNAC54*, *MaNAC13*, *MaNAC34* and *MaNAC58* during the transition from green to red fruit stages indicates their potential roles in fruit pigmentation or taste. Conversely, the decreased expression levels of some other *MaNAC* genes at the purple-fruit stage indicate that they may be primarily involved in early fruit development. For tissue-specific expression pattern, we focused on the OsNAC7 subfamily, which has been reported to act as the master switch in the transcriptional regulation of secondary wall biosynthesis in *Arabidopsis* [46,47,48] and poplar [49,50]. The expression preference of *MaNAC15* and *MaNAC45* in woody tissue are in line with their *Arabidopsis* orthologs *VND7* and *NST1* as the key players in xylem vessel and fiber differentiation [46,51]. Notably, *MaNAC70* and *MANAC71* displayed much higher expression in leaf and bark, suggesting that these two genes might be essential for the secondary cell wall biosynthesis in non-woody tissues. These results of characterized members of the mulberry NAC family substantiate the notion that NACs of the same subfamily from different species conserve similar functions and expression patterns.

NAC transcription factors have been extensively studied for their role in drought tolerance in various plant species [52,53,54]. The differential expressions of *MaNAC*s demonstrate their important roles in response to water deficit. For instance, GWHPDOOT023053 (*MaNAC44*) was strongly induced by drought and recovered back upon rewatering, indicating that it could also act as a transcriptional activator in ABA-mediated dehydration response as its ortholog *ANAC072* (*RD26*) [55]. Similarly, *MaNAC67* displayed opposite responsive patterns in cambium and roots under drought. Its orthologous genes *ANAC071* (At4g17980) and *ANAC096* (At5g46590) in *Arabidopsis* have been reported to work redundantly in the conversion from differentiated cells to cambial cells in wound tissues [56]. ANAC096 can directly interact with ABF2 to synergistically activate RD29A transcription in adaptation to dehydration and osmotic stress [57]. *MaNAC12* is suggested to potentially regulate tapetal development and pollen maturation [58], while its responsiveness in cambium and roots to drought indicates that *MaNAC12* might also play an important role in drought tolerance. Such expression patterns reveal a functional divergence of MaNAC TFs across different tissues [59]. Notably, *MaNAC74* maintained significantly higher expression levels even after rewatering, suggesting its potential role in long-term drought memory. A recent study in soybean also highlights the involvement of NAC TFs in drought memory, where certain NAC TFs were shown to be highly induced in primed plants compared to the unprimed group [60]. However, MaNAC74 was not well categorized into any known subfamilies and its specific functions remain to be investigated. Additionally, we found that overexpression of *MaNAC69*, which is the ortholog of *NST3* in *Arabidopsis*, significantly enhanced drought tolerance. The underlying regulatory mechanisms of this enhancement need further investigation. 

NAC TFs play an important role in the battle against various pathogens [61]. In wheat (*T. awstivum*), 146 *TaNAC* genes were affected by several major fungal pathogens [62]. Twenty-six *HaNAC* genes were differentially expressed in response to sclerotinia head rot caused by *Sclerotinia sclerotiorum* in sunflower (*Helianthus annuus*) [63]. In this study, 20 *MaNACs* were responsive to *C. shiraiana* infection, among which *MaNAC32* showed the strongest upregulation, with approximately 200-fold change. MaNAC32 is the ortholog of JUB1 (JUNGBRUNNEN 1), a core element of the GA-brassimosteroid (BR)-DELLA regulatory module, which negatively regulates the defense response against the bacterial pathogen *Psedomonas syringae* pv. *tomato* DC3000 [64]. TaJUB1-L in wheat might be a positive regulator in resistance for leaf rust pathogen via cytosine methylation at 3′UTR [65]. The specific function of *MaNAC32* should be explored further to uncover its role in *C. shiraiana* resistance in mulberry.

## 4. Materials and Methods

### 4.1. Plant Materials and Stress Treatment

Healthy branches of *M. atropurpurea* cv. Zhongshen1 collected from the National Mulberry Germplasm Resource Bank (Zhenjiang, China) were artificially induced to root as described previously [66]. The rooted cuttings were subsequently transplanted in the pots or the field and cultivated with adequate management. The leaves, developing phloem, bark, developing xylem, wood, roots and fruits at four different developmental stages (S0, inflorescence; S1, green fruits; S2, reddish fruits; S3, purple fruits) were collected from saplings in the field [67]. All of the above samples were ground into fine powder in liquid nitrogen with a mortar and pestle and stored at −80 °C for further analysis. 

Two individual drought experiments on cultivar Zhongshen1 were conducted as reported in our previous studies. The progressive drought stress was achieved by completely withholding water irrigation for 15 days on 3-month-old potted saplings [23]. The other was implemented on 6-month-old potted saplings by reducing the water supply to sustain a consistent ca. 15% of saturated soil water content for 21 days then rewatering to 80% for recovery for 9 days [68]. For sclerotiniose response analysis, healthy and diseased fruits were collected in May when *Ciboria shiraiana* infection was naturally prevailing in mulberry plantations [69]. The roots, stem cambium and fruits of saplings from the above experiments were harvested for transcriptomic analysis. For each treatment, two samples were equivalently mixed to form one biological replicate, and three biological replicates were used in total. Cambium sampling was performed by scraping the white glutinous tissue attached to the xylem and phloem with a scalpel.

### 4.2. Identification of Mulberry NAC

The genome sequence of *M. atropurpurea* cv. “Zhongshen1” (accession number: GWHDOOT00000000) was downloaded from the Genome Warehouse (GWH) of the China National Center for Bioinformation (https://ngdc.cncb.ac.cn/gwh/, accessed on 11 July 2024). The genome data of *A. thaliana* were downloaded from the TAIR (https://www.arabidopsis.org/, accessed on 11 July 2024) database. The Hidden Markov Model (HMM) profile of the featured NAC domain (PF02365) retrieved from the pfam database (http://pfam.xfam.org/, accessed on 11 July 2024) was used to conduct HMM search against the whole annotated protein database with an E-value cutoff of 1 × 10^−8^ using TBtools software (version 2.1.04) with default parameters [70]. The obtained candidate NAC proteins were further verified by using the CD-search online program in the Conserved Domain Database (CDD) (https://www.ncbi.nlm.nih.gov/cdd, accessed on 11 July 2024). The remaining protein sequences with the complete NAC domain were aligned using ClustalW and MEGA X. The physicochemical property analysis of the NAC gene family was conducted using Tbtools (version 2.1.04). The predicted NAC proteins in *M. atropurpurea* and *A. thaliana* were noted as “MaNAC” and “AtNAC,” respectively.

### 4.3. Phylogenetic Tree Construction, Chromosomal Localization and Collinearity Analysis

The NAC protein sequences of *M. atropurpurea* and *A. thaliana* were aligned using the algorithm MUSCLE in MEGA 7.0, and a circular phylogenetic tree was constructed using the Neighbor-Joining (NJ) method in MEGA 7.0 with 1000 bootstrap replicates. We mapped the *MaNAC* gene chromosomal positions using TBtools software (version 2.1.04). The collinearity and gene replication analysis were performed with Circle Gene View and Advanced Circos in Tbtools (version 2.1.04).

### 4.4. Bioinformatic Analysis of MaNACs

Exon–intron structures of *MaNAC* genes were displayed based on the genome sequence and its annotation file using Gene Structure View assembled in Tbtools (v2.142). Conserved motifs were identified in the MaNAC proteins using the online program MEME (http://meme-suite.org/tools/meme, accessed on 11 July 2024) with a maximum of 20 motifs and a range of motif widths from 6 to 50 as parameters. The 2000 bp sequences upstream of the start codon of *MaNAC* genes were submitted to the online resource PlantCARE (http://bioinformatics.psb.ugent.be/webtools/plantcare/html/, accessed on 11 July 2024) for cis-element analysis. The output file was used to illustrate the cis-element distribution in promoters of *MaNAC* using Tbtools (version 2.1.04).

### 4.5. Expression Profiling of MaNAC Based on Transcriptome Analysis

The expression patterns of *MaNAC* genes were extracted from the transcriptome data of healthy mulberry fruits at four different developmental stages, fruits infected or uninfected with *C. shiraiana*, and cambium and roots exposed to drought stress. Detailed information for the transcriptome of mulberry fruit sclerotiniose was reported in our previous study [69], and the transcriptome processing and analysis of cambium in response to 15% drought stress and rewatering is well documented in our recent publication [68]. For the root samples from the progressive drought experiment and the fruit samples of different developmental stages, RNA sequencing was performed on the BGISEQ-500 platform. FPKM (fragment per kilobase of transcript per million mapped reads) value was calculated for gene expression levels.

### 4.6. qRT-PCR Analysis

For genes of the *OsNAC7* subfamily, the total RNA was extracted from the frozen powder of plant materials including the leaves, developing phloem, bark, developing xylem, wood and roots according to the instructions of a plant RNA extraction kit (R6827, Omega Bio-teck, Norcross, GA, USA). The first-strand cDNA was synthesized using a PrimeScript™ RT reagent kit with gDNA Eraser (RR047A, Takara Bio, Kyoto, Japan) following the manufacturer’s protocol. qRT-PCR was performed using TB Green^TM^ Premix Ex Taq II (RR820A, TaKaRa Bio, Kyoto, Japan) on an ABI StepOnePlus™ Real-Time PCR System (Applied Biosystems, Massachusetts, USA) as described previously [71]. Three technical replicates for each of the three biological replicates were used for each organ. For the expression of *MaNAC69* in transgenic *Arabidopsis*, total RNA from rosette leaves was extracted and qRT-PCT was conducted with three biological replicates. The relative expression levels were calculated by the 2^−∆∆Ct^ method [72]. The mulberry actin gene was used as the reference gene, and the gene-specific primers of the six members in the OsNAC7 subfamily are shown in Table S5. SPSS19.0 was used to perform *t*-test and ANOVA; *p* < 0.05 was considered significant. GraphPad Prism8.0 was used to visualize the qRT-PCR results.

### 4.7. Subcellular Localization

The full-length CDS of *MaNAC15* and *69* was cloned (refer to Table S5 for primer information) and ligated into the multiple cloning sites of pCambia1300 expression vector recombined with a CaMV 35S promoter and GFP flag, resulting in the formation of the recombinant pCambia1300-35S-MaNAC69-GFP and pCambia1300-35S-MaNAC15-GFP plasmids. For subcellular localization analysis, the plasmids were fused with an mCherry gene containing a nuclear localization signal (NLS). The resulting recombinant plasmid was transformed into tobacco (*Nicotiana benthamiana*) cells using the *Agrobacterium tumefaciens* strain GV3101 via Agrobacterium-mediated transient transformation [73]. The bacterial suspension was injected into the abaxial side of 4-week-old tobacco leaves. After 3 days of dark culture, mCherry fluorescence was observed under a Zeiss LSM 900 laser confocal microscope (Magdeburg, Germany) to determine nuclear localization. Additionally, GFP fluorescence in the leaves was also observed under the microscope after the same dark culture period.

### 4.8. Functional Analysis of MaNAC69 in Arabidopsis in Response to Drought

Recombinant plasmids used for subcellular localization of MaNAC69 were transformed into *Agrobacterium tumefaciens* strain GV3101. The floral dip method was adopted to obtain the transgenic *Arabidopsis* seeds and further positive seedlings were screened using Hygromycin B (20 mg/L) [74]. The transgenic seedlings were further confirmed by qRT-PCR to detect the overexpression of *MaNAC69* in *Arabidopsis* compared to WT (Col-0). Briefly, three positive lines (OE2, OE6 and OE10) of the T2 generation and the wild type were stratified at 4 °C for 3 days on agar plates containing half-strength Murashige and Skoog medium (1/2 MS). Then, they were cultivated in a controlled growth chamber with 22/18 °C day/night temperature, 60% relative humidity, 16 h/8 h light/dark cycle and 200 μmol m^–2^ s^−1^ light intensity for 10 days. Subsequently, the seedlings were transplanted in plastic pots (7 cm × 7 cm × 9 cm) filled with soil and vermiculite (*v*/*v* = 1:1) and cultivated for 10 days, with 4 seedlings per pot. Drought stress was inflicted on 21 pots per line by withholding water for 7 days, followed by 2 days of rehydration. The control group with 3 pots was watered regularly [17].

## 5. Conclusions

In summary, this study provides a comprehensive analysis of the MaNAC gene family in *M. atropurpurea*, including their phylogenetic relationships, conserved motifs, cis-elements, temporal expression in fruit development and stress responses to drought and sclerotiniose. A set of candidate genes that are potentially associated with the above biological processes were identified. Particularly, several members of the OsNAC7 subfamily were functionally analyzed for their role in drought tolerance. Future studies should focus on the functional characterization of key *MaNAC* genes to uncover their roles in mulberry growth and stress adaptation, thereby facilitating future breeding of drought- and sclerotiniose-resistant germplasm or cultivars.

## Data Availability

The original contributions presented in this study are included in the article/Supplementary Materials. Further inquiries can be directed to the corresponding author(s).

## Supplementary Materials

The following supporting information can be downloaded at: https://www.mdpi.com/article/10.3390/plants14081179/s1, Figure S1: Multiple-sequence alignment of the amino terminus from the 79 MaNAC proteins. The locations of the five highly conserved NAC subdomains (A–E) were indicated; Figure S2: The 20 motifs identified in *MaNAC* genes; Table S1: Basic characteristics of NAC family in *M. atropurpurea*; Table S2: The number of exons in *MaNAC* genes; Table S3: Cis-elements in the promoter of *MaNAC* genes; Table S4: The functional grouping of cis-elements and the proportion of each group; Table S5: The primer sequences used in this study.
