# Supplementary figures and images for: Genome-Wide Analysis of NAC Transcription Factor Gene Family in Morus atropurpurea
Source: Plants (Basel). 2025 Apr 10;14(8):1179. doi: 10.3390/plants14081179 (PMC12030528; doi:10.3390/plants14081179)

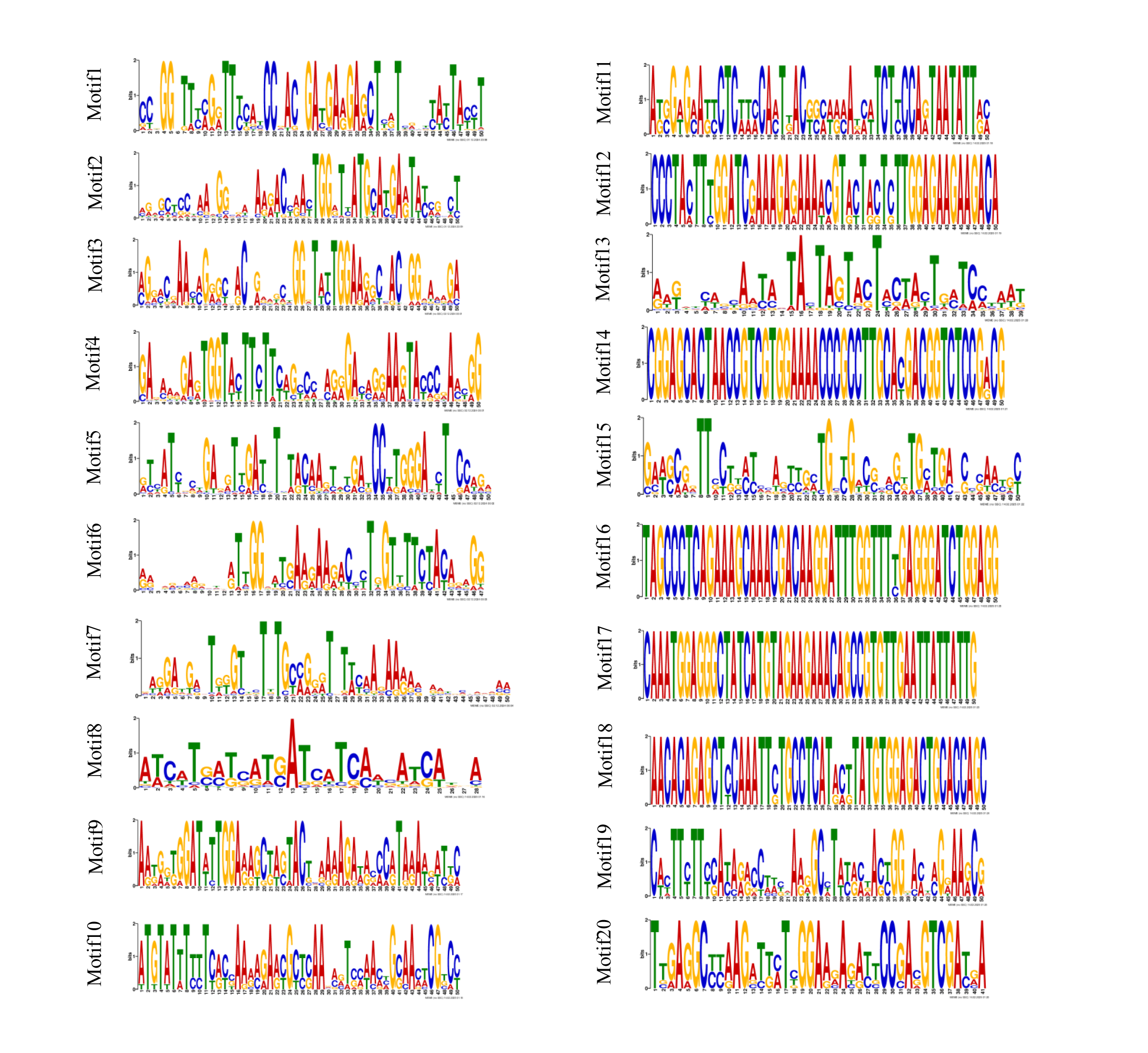

Supplement: Supplementary file 1 [file plants-14-01179-s001.zip › Figure S2.tif]
